# A Study on the Surface Quality and Damage Properties of Single-Crystal Silicon Using Different Post-Treatment Processes

**DOI:** 10.3390/mi15010145

**Published:** 2024-01-17

**Authors:** Wei Li, Fangyuan Zha, Bo Fu, Yanglong Li, Jiazhu Duan, Ziyou Zhou

**Affiliations:** 1 Institute of Fluid Physics, China Academy of Engineering Physics, Mianyang 621900, China; vleefoxtrot@126.com (W.L.); 17863909069@163.com (F.Z.); jesseliyl@163.com (Y.L.); jzduan_oe@163.com (J.D.); 2 School of Materials Science and Engineering, Central South University, Changsha 410083, China; 193102061@csu.edu.cn

**Keywords:** silicon, etching, weak absorption, laser damage

## Abstract

Detecting subsurface defects in optical components has always been challenging. This study utilizes laser scattering and photothermal weak absorption techniques to detect surface and subsurface nano-damage precursors of single-crystal silicon components. Based on laser scattering and photothermal weak absorption techniques, we successfully establish the relationship between damage precursors and laser damage resistance. The photothermal absorption level is used as an important parameter to measure the damage resistance threshold of optical elements. Single-crystal silicon elements are processed and post-processed optimally. This research employs dry etching and wet etching techniques to effectively eliminate damage precursors from optical components. Additionally, detection techniques are utilized to comprehensively characterize these components, resulting in the successful identification of optimal damage precursor removal methods for various polishing types of single-crystal silicon components. Consequently, this method efficiently enhances the damage thresholds of optical components.

## 1. Introduction

Optical component processing typically involves grinding, polishing, shaping, and other abrasive-based processing technologies. These processes generate small brittle/plastic cracks, scratches, and other physical defects on the surface of the components. These defects not only affect the mechanical properties of the optical element, but also greatly impact its optical properties. Additionally, residual chemical contamination from polishing abrasives and other chemical contaminants can cause laser-induced damage in high-energy/high-power laser systems, resulting in optical component failure [1,2,3,4]. In high-energy laser systems, cracks, contamination, and other factors that cause laser-induced damage to optical elements constitute damage precursors. The distribution characteristics of weak absorption on the surface of fused silica optics post-treated with different processes and 355 nm laser-induced damage properties were investigated by Shi et al [5]. The correlation between defects with different absorption levels and the damage performance of fused silica surfaces was systematically investigated. It is shown that there is a significant correlation between the density of defects with absorption above 2 ppm and the damage performance. Feng Shi studied the evolution of several nanoscale damage precursors in shallow IBE, and found that IBE can eliminate the polished redeposited layer and weaken the surface densification. It is important to determine the intrinsic properties of nanoscale damage precursors in terms of surface laser damage resistance [5,6,7,8,9]. Damage precursors originate from the processing, handling, coating, and other processes of optical components. Therefore, it is necessary to monitor and control damage precursors throughout the entire processing of optical components to ensure that they meet the requirements of high-energy laser systems [10,11]. Silicon-based materials are widely used in infrared thermal imaging systems due to their good spectral properties in the mid-infrared region. In high-power continuous laser systems, silicon-based materials are commonly used as cavity mirrors, mirrors, and other optical components due to their excellent integrated thermal performance ratios and mid-infrared optical properties. The quality of the mirrors directly determines the performance of the laser system [12,13,14,15]. With the development of high-power lasers, the lenses in laser systems need to withstand higher and higher power densities, and the most important limiting factor for the performance of the lenses is the damage precursor.

**Figure 1:**
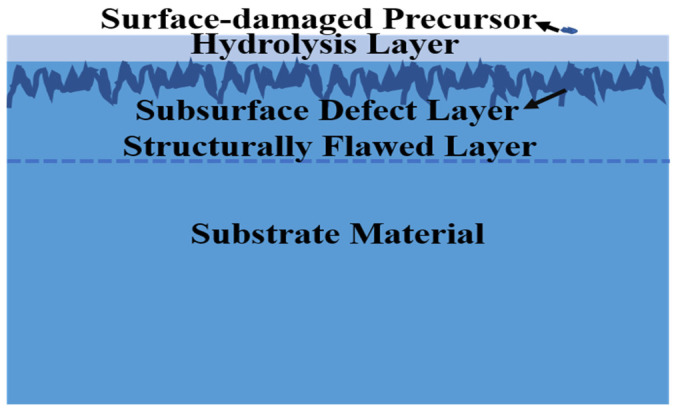
Surface defects and subsurface defects in single-crystal silicon components after processing.

During the grinding and polishing of silicon lenses, monocrystalline silicon, being a brittle material, is prone to cracks, impurities, and other defects [16]. Figure 1 illustrates the common defect distribution of optical materials after grinding and precision polishing. Surface and subsurface cracks, as well as impurity metal elements, may be present. These defects can be suppressed as the level of processing increases, and a layer of hydrolysis forms below the surface of the element. In brittle materials such as monocrystalline silicon, the hydrolysis layer is a layer with a refractive index comparable to that of silicon, but amorphous [17,18,19,20]. Beneath the hydrolysis layer is a layer of subsurface crushing defects, which includes many scratches and cracks due to grinding and polishing, as well as a layer of defects enriched by polysilicon. Further below is a structural defect layer brought to the substrate by the grinding and extrusion process [21].

Extensive research has established a consensus that laser-induced defect sites reduce the laser damage resistance of optical materials [22,23,24,25]. In continuous high-energy laser systems, the difference between the light absorption caused by defects and the body material creates a significant stress mismatch at the defect site. When the aberration caused by the stress mismatch exceeds the intrinsic cracking strength of the material, laser-induced damage occurs [26,27]. Surface and subsurface defects in optical elements can extend to affect functional multilayers, leading to damage due to nodule-type defects during intense laser loading. Additionally, the presence of defects on the surface of an optical element when irradiated by an intense laser leads to a large amount of heat generation under prolonged, high-energy laser irradiation of the element. Surface and subsurface damage precursors are highly susceptible to stress differences between the defects and the body material under the action of the thermal field. Such stress differences can lead to deformation or even damage in the local region of the element [28,29,30,31]. Therefore, the existence of these defects can significantly reduce the damage resistance of the film layer and even the entire optical element. It is, therefore, necessary to establish a means of detecting damage from surface/subsurface laser-induced thermotropic damage mechanisms in optical elements.

Furthermore, even after polishing optical components, micro-nanometer damage precursors may still exist on the surface and subsurface. The use of appropriate detection means is necessary to identify these precursors, and their removal is an important scientific issue. In this study, dry etching and wet etching technologies were employed to remove damage precursors from single-crystal silicon optical components. Laser scattering detection technology and photothermal weak absorption detection technology were used to assess the state of the components before and after treatment, ultimately leading to the identification of the optimal treatment method. Moreover, dry etching and wet etching technologies were found to effectively enhance the damage threshold of optical components.

## 2. Experimental

In our study, monocrystalline silicon optics were selected for each of the three different processing processes. All monocrystalline silicon crystals have an orientation of 100, a diameter of 50 mm, and a thickness of 5 mm. Bonnet polishing (BP) allows for uniform polishing over large areas and improves the surface finish of optical elements, but for high surface roughness and raised defects, multiple passes may be required to achieve the desired smoothness. However, for defects with high surface roughness and raised surfaces, multiple polishing passes may be required to achieve the desired smoothness. Chemical–mechanical polishing (CMP) is capable of treating surfaces of various shapes and edges, and is effective in removing small defects such as scratches and oxidized layers. However, the polishing process is more complex and requires control of the concentration, flow rate and time of the polishing solution. Magnetorheological polishing (MRF) can be performed on surfaces with unconventional shapes such as spherical and aspherical surfaces. However, the preparation of the polishing solution is more complicated and requires the configuration of special magnetorheological fluids. We provide the corresponding processes for each single-crystal silicon optical element in Table 1.

We use two different etching methods to etch damage precursors on the surface of optical elements. The first is a dry etching technique, for which we choose sulfur hexafluoride gas (SF6) and helium (He) as etching gases for the etching process. In the etching process, in general, increasing the gas flux ratio can increase the ion density and particle energy, thus increasing the etching rate. However, a too high gas flux ratio may lead to an increase in the collision between ions and thus decrease the etching rate. In addition, the etching rate increases with increasing pressure in the RIE process, and gas molecules collide with active particles in the plasma to produce an etching reaction. Increasing the pressure increases the concentration of gas molecules and the frequency of collisions, thereby increasing the etching rate. However, when the pressure is too high, the collision frequency in the plasma may be too high, resulting in the saturation or reduction of the etch rate, and possibly even “over-etching”. In addition, an increase in etching time leads to deeper etching, but this relationship depends to some extent on the etching gas used, the process parameters, and the material being etched. Therefore, based on the above analysis, we chose the following three research programs to study the dry etching process, and the specific etching process is shown in Table 2.

Prior to dry etching, we cleaned the surface area of the optical element using organic solvents to remove dust, oil, and impurities [32,33]. During the dry etching process, we employed three etching processes to remove the damage precursors on the surface of the optical element, based on previous etching experience. Table 2 outlines the three processes used. We observed that after etching, the surface of the sample appeared whitish, and the roughness increased significantly in method 1. This was mainly due to the excessive etching power and etching time. In method 2, the sample surface was not mirror-like after etching, but the roughness also increased significantly. In method 3, we found that the sample was mirror-like after etching, and the roughness did not change significantly. Therefore, for this study, we utilized option 3 to dry etch the samples.

In addition, we also used a wet etching process to remove the damage precursors on the surface of the optical elements, and the etching process is shown in Table 3. Before wet etching, we first used HF acid etching for 1 min to remove the surface oxide layer. For the three etching processes in Table 3, we found that there was no obvious effect on the sample surface after the completion of etching in approach 1, the sample surface was mirrored after the end of etching in approach 2, and the etching efficiency was very slow. The sample surface was mirrored after the end of etching in approach 3, and the etching efficiency was significantly improved. In the etching process, KOK and silicon can produce a chemical reaction to remove the sample, and isopropanol can be used to enhance the etching rate. Therefore, for the study in this paper, we used approach 3 for wet etching the sample.

In order to complete the study, we mainly adopted the following two methods of testing the damage precursors of optical elements. Firstly, we used a laser scattering detection device to test the loss precursors on the surface of the optical element. The test device’s optical path was built for this purpose; the laser in the test optical path had an output wavelength of 532 nm, a light-sensitive area of 0.6 mm × 0.6 mm, and a resolution of 512 × 512. The main test principle is that when the beam passes through the inhomogeneous media, part of the beam will deviate from the original direction, and there will be dispersed propagation; the light from the side can also be seen, so that the defects on the surface of the optical components and scratches can be seen [34,35]. Secondly, for the photothermal weak absorption test, the optical path was built for this purpose. For the test optical path, we selected a 3.8 μm laser as the pump light, a 632.8 nm laser for the detection of light on the photothermal weak absorption test, and a test range of 5 mm × 5 mm. Using the results of the weak absorption test, the weak absorption data can form a kind of relative ratio of the pump light irradiation area to the photothermal weak absorption produced. This weak absorption causes a change in the local region of the material, and this change modulates the detected light that also passes through the region [36,37,38]. The weak absorption data are thus measured as the ratio between the absorption and the intensity of the detected light [39,40].

**Figure 2:**
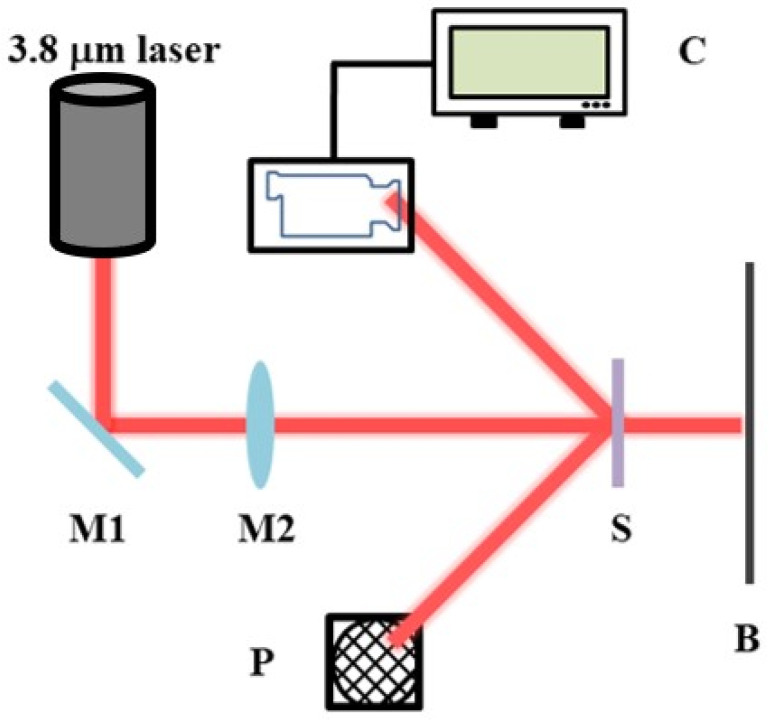
Schematic diagram of the damage test light path. The laser is 3.8 μm, M1 is an all-reflective mirror, M2 is a focusing lens set, S is a sample, C is an online damage-monitoring CCD, P is a reflected light collection energy meter, and B is a transmitted light collection bin.

For the characterisation of the final damage test, we used the optical path shown in Figure 2. The laser outputs a 3.8 μm laser, which is focused by a lens and then irradiates the optical element. Prior to the laser damage threshold test, the laser output power and spot size were measured, the sample test spot was placed in the laser light path and irradiated using individual laser pulses of different energy densities. For each selected pulse energy, at least five test points were irradiated and recorded, and the actual pulse energy used for each test point was recorded using a beam diagnostic device. The condition (damaged or undamaged) of the sample after laser irradiation was also recorded. This test was repeated for other pulse energies. The range of pulse energies used should be wide enough to ensure that the minimum energy value will not cause damage to any of the sample test points. The maximum energy value is capable of damaging every sample test point, and damage threshold data are obtained using the damage probability method [41,42].

## 3. Result and Discussion

### 3.1. Surface Morphology of Un-Etched Samples

**Figure 3:**
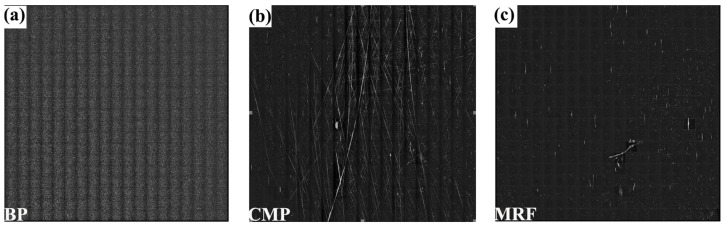
Scattering test plots of samples from different polishing processes. (**a**–**c**) Bonnet-polishing process, chemical–mechanical polishing process, and magnetorheological polishing process, in that order.

Scattering test techniques enable the detection of micro and nano-level surface defects of optical components [43,44]. As shown in Figure 3, the bonnet-polished sample (Figure 3a) exhibited numerous scattering points, and the surface quality was poor, with more pockmarks and scratches. The CMP-polished sample (Figure 3b) also had more scratches and pockmarks on the surface, but the number of pockmarks was significantly reduced compared to the bonnet-polishing process. In Figure 3c, the number of scratches in the MRF-polished samples is significantly lower than that in the CPM-polished samples, and the number of pockmarks is less than that of the previous two processes, with a significant improvement in the surface quality of the samples. By comparing these three processes, we conclude that the MRF process can effectively remove scratches and pockmarks on the surface of the sample, and repair the roughness and surface shape of the sample.

**Figure 4:**
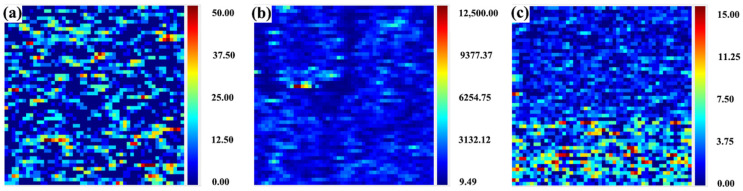
Photothermal weak absorption test plots of samples with different polishing processes. (**a**–**c**) bonnet-polishing process, chemical–mechanical polishing process and magnetorheological polishing process, in that order.

Figure 4 presents the photothermal weak absorption test results of samples with different polishing processes. The photothermal weak absorption technique enables the detection of subsurface defects of optical elements. As shown in Figure 4, the distribution of subsurface defects of bonnet-polished samples is more extensive and covers a larger area. This distribution is mainly due to the bonnet-polishing process, which uses flexible bonnets and abrasive materials to smooth out scratches or defects on the surface of the optical element [45,46]. However, craters and deep scratches cannot be removed, resulting in a large area of weak absorption test results. This also explains the reason for the high number of pockmarks in the scattering test [47]. CMP polishing involves the addition of abrasive materials in the chemical solution to polish the optical element. However, the polishing solution contains elements such as cerium, which introduces metal elements and results in very highly weak absorption values. The MRF polished samples have fewer subsurface defects compared to the previous two processes, and their distribution range is smaller than that of the bonnet-polished samples. Table 4 provides the specific values of weak absorption for these three polishing processes.

### 3.2. Sample Morphology after Etching

**Figure 5:**
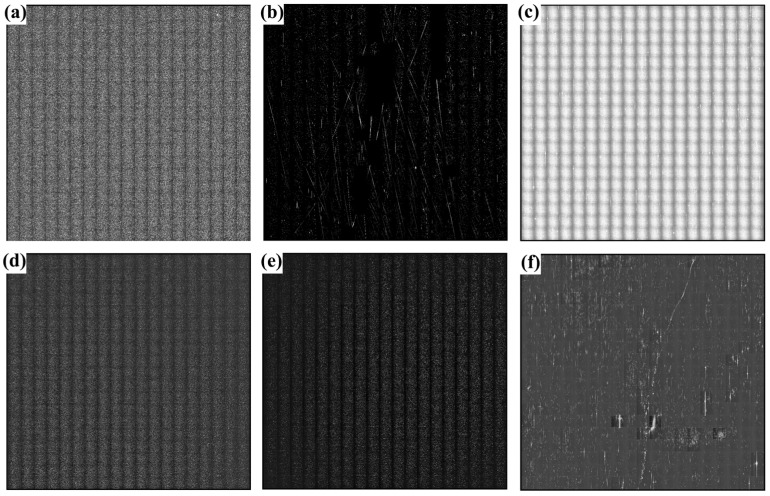
Scattering test plots of the samples with different processes after wet and dry etching. Panels (**a**–**c**) show the dry etching of samples from the bonnet-polishing process, chemical–mechanical polishing process, and magnetorheological polishing process, respectively. Panels (**d**–**f**) show the wet etching of samples from the bonnet-polishing process, chemical–mechanical polishing process, and magnetorheological polishing process, respectively.

We selected samples after the bonnet-polishing, CMP, and MRF polishing processes, and performed dry etching and wet etching for each process to treat the samples. The results of the scattering test are presented in Figure 5. Regardless of the polishing process used, a large number of point features were observed on the surface of the samples after each cleaning, with a significant increase in the number of scratches and pockmarks, and an increase in roughness. These point defects may be caused by impurities in the polished redeposition layer of the samples [48]. The number of scattered points on the surface of the samples after wet etching was smaller than that of the samples after dry etching. The results demonstrate that both dry etching and wet etching techniques can effectively remove defects on the surface of the optical element and expose defects embedded in the subsurface. It was also shown that the laser-induced scattering imaging technique can be effective in the non-destructive detection of single-crystal silicon surface defects.

**Figure 6:**
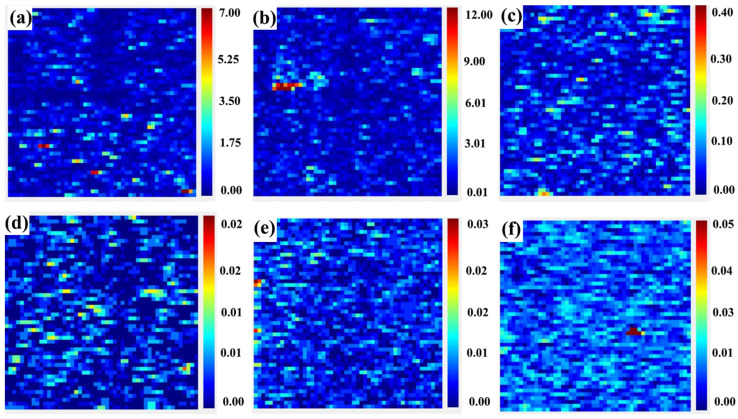
Photothermal weak absorption test plots of samples with different processes after wet and dry etching. Panels (**a**–**c**) show the dry etching of samples from the bonnet-polishing, chemical–mechanical polishing, and magnetorheological polishing processes, respectively. Panels (**d**–**f**) show the wet etching of samples from the bonnet-polishing, chemical–mechanical polishing, and magnetorheological polishing processes, respectively.

Figure 6 presents the photothermal weak absorption detection results. It can be observed that there are numerous discrete absorption defects on the surface of all the samples. The highest value of photothermal weak absorption for the wet-etched sample is one order of magnitude lower than that of the dry-etched sample. Combining the photothermal test results of the unetched samples, we found that the average and peak values of weak absorption for the samples polished by the MRF process are lower than those of the samples processed by the other two processes. The samples processed by the MRF process have a more uniform distribution of impurity metal elements on the subsurface, and fewer defective spots or pits on the surface. For samples of the same process, wet etching is superior to the other two processing process. Dry etching causes a certain change in the surface quality of the samples, but due to the possibility of contamination of the samples during the etching process, the effect of post-processing is not very significant. Therefore, we conclude that for silicon optical elements, wet etching can be the optimal means to effectively remove defects present on their surfaces and subsurfaces. The results demonstrate that the weak absorption detection technique is good for non-destructive detection of subsurface defects in single-crystal silicon optical devices [49]. However, due to the relatively macroscopic measurement results, it is difficult to identify the morphology or shape of the defects. This signifies a potential area for future research.

### 3.3. Damage Threshold and Morphology

**Figure 7:**
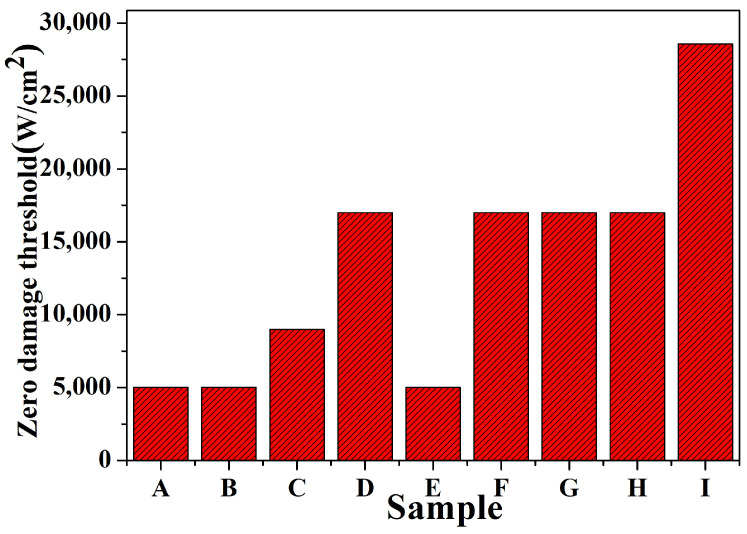
Statistical plots of zero-damage thresholds for samples with different processing techniques and for samples after etching. A–C are bonnet-polished samples, D–F are CMP-polished samples, and G–I are MRF-polished samples. B, E, and H are dry-etched samples. A, D, and G are wet-etched samples. C, F, and I are wet-etched samples.

During our damage testing process, we set a requirement that the test energy step should not be less than 5, and each energy step test point should be 5. We recorded the highest energy density when no damage occurred and the energy density when damage occurred. We then calculated the average value of these two values for each point, which represents the damage threshold of the point. We averaged the damage threshold of all the test points to obtain the damage threshold of the sample. The sample testing environment was a class 10,000 clean room with a humidity of 45.6%. The laser-emitting surface was used as the sample-testing surface, and acetone and isopropyl alcohol were used to clean the sample surface before testing. During the damage test, the laser output wavelength was set to 3.8 μm, the pulse width was 25–35 ns, and the mode was TEM00. The laser energy was Gaussian-distributed, and the laser had to be warmed up for at least 15 min before the official test to ensure the stability of the output. The results of the damage threshold test are presented in Figure 7.

Based on the above study, we first conducted damage tests on the original samples to determine the damage thresholds of different polishing processes under the same conditions. Second, we used different methods to treat samples with the same polishing process and then determine how the different processes improve the damage threshold of the sample. Finally, we compare the results to determine which polishing process has the highest damage threshold under certain conditions. The test results are actual test values. It was found that the zero-probability damage threshold and average damage threshold of the MRF-treated samples were better than those of the bonnet-polished and CMP-polished samples; moreover, for the samples of the same process, the damage threshold and the average damage threshold of the samples treated by wet etching were better than those of the samples cleaned by dry etching and organic solvents, which is in agreement with the results of the photothermal weak absorption test. However, for the CMP-polished samples, the damage threshold enhancement was not significant due to the residual metallic impurity elements on the surface of the samples themselves, which may expose these defects in the subsequent etching. Among the three processes for the same sample, the sample quality of the MRF-polished polished sample is better than that of the bonnet-polished and CMP-polished samples, with a more uniform distribution of metal impurity elements on the surface, fewer surface craters, and better results of the damage threshold test; for the same process, the wet-etched sample has the best surface quality, which effectively removes defects on the surface and subsurface of the monocrystalline silicon element.

**Figure 8:**
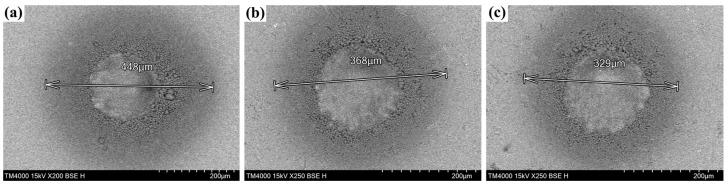
Damage crater morphology of bonnet-polished samples. (**a**) primary sample; (**b**,**c**) are the samples after dry etching and wet etching, respectively.

Figure 8 shows the morphology of the damage crater observed with a scanning electron microscope after the damage test. The size of the damage pits is marked in the figure. Our observation indicates that the damage occurred on the front surface of the sample, which is the incident surface of the laser. By comparing the damage morphology after different etching processes, we found that the optical element in the center of the laser spot underwent melting, followed by the production of solid particles around the melting area due to the process of melting and cooling after high-temperature treatment. The outermost edges also produced some spattering of the material during the laser action. The size and depth of the damage pits were significantly smaller in the etched sample than in the unetched sample. In summary, the damage is mainly due to the thermal melting phenomenon caused by the temperature in the center of the optical element during the laser-loading process, which exceeds the melting point of the material itself [50].

## 4. Conclusions

Through the implementation of a laser-scattering test and the photothermal weak absorption technique, we have effectively established a correlation between damage precursors and the resistance of materials to laser damage. In addition, we employed photothermal absorption level as a crucial parameter to determine the damage resistance threshold of optical elements. In this study, we investigated the damage precursors of samples with different processing techniques, using the laser-scattering test and photothermal weak absorption test to compare the characteristics of different processing techniques. Following that, we utilized dry etching and wet etching techniques to effectively eliminate the damage precursors. Subsequently, these precursors were characterized using a range of testing techniques. We successfully obtained optimal damage precursor removal methods for polishing different types of single-crystal silicon components and determined the relationship between the effects of different damage precursor removal methods on damage performance. These findings have the potential to significantly enhance the damage threshold of optical components.

## Figures and Tables

**Table 1:** Three sample-polishing processes.

Sample	Polishing Process
A	Bonnet polishing
B	Chemical mechanical polishing
C	Magnetorheological finishing

**Table 2:** Dry etching parameters.

Approach	SF_6_ (sccm)	He (sccm)	Power (W)	Time (min)
1#	10	100	400	30
2#	20	20	100	25
3#	30	30	200	15

**Table 3:** Wet etching parameters.

Approach	Etching Solution	Time (min)
1#	KOH	30
2#	KOH	10
3#	KOH + C_3_H_8_O	15

**Table 4:** Weak absorption values for different polishing processes.

Sample	Max	Min	Average
A	35.8	0.00	7.21
B	4307.31	9.49	1346.20
C	10.81	0.00	2.59

## Data Availability

Data are contained within the article.
